# Hyperspectral image compressed processing: Evolutionary multi-objective optimization sparse decomposition

**DOI:** 10.1371/journal.pone.0267754

**Published:** 2022-04-29

**Authors:** Li WANG, Wei WANG

**Affiliations:** Department of Electronic Engineering, Xi’an Aeronautical University, Xi’an, Shaanxi, China; Universidad de Guadalajara, MEXICO

## Abstract

In the compressed processing of hyperspectral images, orthogonal matching pursuit algorithm (OMP) can be used to obtain sparse decomposition results. Aimed at the time-complex and difficulty in applying real-time processing, an evolutionary multi-objective optimization sparse decomposition algorithm for hyperspectral images is proposed. Instead of using OMP for the matching process to search optimal atoms, the proposed algorithm explores the idea of reference point non-dominated sorting genetic algorithm (NSGA) to optimize the matching process of OMP. Take two objective function to establish the multi-objective sparse decomposition optimization model, including the largest inner product of matching atoms and image residuals, and the smallest correlation between atoms. Utilize NSGA-III with advantage of high accuracy to solve the optimization model, and the implementation process of NSGA-III-OMP is presented. The experimental results and analysis carried on four hyperspectral datasets demonstrate that, compared with the state-of-the-art algorithms, the proposed NSGA-III-OMP algorithm has effectively improved the sparse decomposition performance and efficiency, and can effectively solve the sparse decomposition optimization problem of hyperspectral images.

## Introduction

Hyperspectral images (HSIs) contain rich spatial geometric information and spectral feature information, which are suitable for target detection and recognition, image classification and other fields [1–3]. The expansion of the application field proposes complete spatial resolution and spectral resolution, needs to reduce the dimensionality [4–6] of hyperspectral data. In the process of dimensionality reduction, how to ensure the integrity of information is a big problem. Furthormore, sharp increase in the amount of information, further brings challenges to data storage and transmission. The traditional Shannon sampling theorem is no longer suitable for large data volume hyperspectral image processing.

Compressed Sensing (CS) theory [7, 8] was proposed in 2006, with the characteristics of directly collecting data information. The essence of compressed sensing theory is to extract as much information as possible from as little data as possible, combine compression and sampling at the sampling end, and reconstruct the original signal with high precision at the reconstruction end. Random sampling is performed at a lower sampling rate than the Nyquist sampling frequency, and reconstruct the original signal with optimization algorithms. The sampling of signals can break through the limitations of the traditional Nyquist law and greatly reduce the amount of data, but its application premise is that the signal meets the sparseness or compressibility conditions.

The first task of applying compressed sensing theory to process hyperspectral images is achieving sparse decomposition. Tensor decomposition are used to take advantage of the low rank and sparsity of images to obtain sparse representation of hyperspectral images, to realize the hyperspectral image unmixing [9], fusion [10] and restoration [11]. Sparse decomposition can also be used for image denoising [12].

Hyperspectral image is a complex signal with multiple structural components, and it is difficult to effectively sparsely represent it using orthogonal transform. Increasing the number of atoms to form a redundant dictionary is conducive to sparse representation of complex signals. Among the many sparse decomposition algorithms, Orthogonal Matching Pursuit (OMP) [13, 14] is a commonly used sparse decomposition algorithm for compressed reconstruction.

The redundant dictionary is used to sparsely represent the hyperspectral image signal, which can improve the accuracy of sparse decomposition, but it also brings about problems. The high complexity of the algorithm poses new challenges to the computational efficiency of the sparse decomposition algorithm. In fact, sparse decomposition is a process of objective function optimization, so we consider using intelligent optimization algorithms to complete the solution.

We construct a multi-objective sparse decomposition optimization model that takes the largest inner product of matching atoms and image residuals, and the smallest correlation between atoms as the optimization goal. Aiming at this multi-objective sparse decomposition optimization model, the multi-objective evolutionary algorithm [15–17] is good choice to solve it. Considering the accuracy and efficiency, the non-dominated sorting genetic algorithm (NSGA-III) [18, 19] is explored to complete the solution of optimization model. An improved sparse decomposition algorithm with the help of OMP and NSGA-III is proposed, denoted as NSGA-III-OMP. The innovation of this paper is, abandoning the traditional optimal atom matching method, choosing the optimal atom selection using the evolution process of NSGA-III, so that the atom describing the complex hyperspectral image can be found more accurately.

The remainder of the manuscript is organized into six sections. In Section 2, the mathematical model, including the Gabor dictionary basis construction and the multi-objective sparse decomposition optimization model are presented. In Section 3, the NSGA-III method is introduced firstly, with its selection operator, crossover operator, mutation operator, and its steps are illustrated. The NSGA-III-OMP is described and the implementation process of algorithm are shown. In Section 4, experimental datasets and evaluation metrices are presented. The parameter selection is described in Section 5, with results and comparisons shown in Section 6. Section 7 is about conclusion.

## Multi-objective sparse decomposition optimization model

### Construction of redundant dictionary

The sparse representation ability of single orthogonal basis cannot satisfy image signals with complex features, therefore, redundant dictionary composed of non-orthogonal atoms is used for sparse decomposition and representation.

Using the Gabor function to generate a redundant dictionary for sparse representation of the signal can ensure the global information of the signal and the intensity of the signal change in any local time. The generation function of the Gabor dictionary is [20, 21],

$$g_{\gamma }\left(n\right)=\frac{1}{\sqrt{s}}win\left(\frac{n-u}{s}\right)\text{cos}\left(\upsilon n+\omega \right)$$ (1)

Where, $win\left(n\right)=e^{-\pi n^{2}}$ is the Gaussian window function, *n* = 0,1,…, *N*, *N* is the signal length, *γ* = (*s*,*u*,*υ*,*ω*) is time-frequency parameter. Let ***D*** = {***g***_*γ*_}_*γ*∈Γ_ denote the redundancy dictionary, in which ***g***_*γ*_ is the atom defined by the parameter group *γ*, Γ is the set of parameter groups. The time-frequency parameters are discretized according to the following methods: $\gamma =\left(a^{\alpha_{1}},\alpha_{2}a^{\alpha_{1}}\Delta u,\alpha_{3}a^{-\alpha_{1}}\Delta \upsilon ,\alpha_{4}\Delta \omega \right)$, whereas, *a* = 2, Δ*u* = 1/2, Δ*υ* = *π*, Δ*ω* = *π*/6, 0 < *α*_1_ ≤ log_2_*N*, $0\leq \alpha_{2}\leq 2^{-\alpha_{1}+1}N$, $0\leq \alpha_{3}<2^{\alpha_{1}+1}$, 0 < *α*_4_ ≤ 12. The Gabor dictionary is highly redundant, and the number of atoms is *N*_atom_ = 52(*N*log_2_ *N* + *N* − 1).

### Sparse decomposition of OMP

The basic idea of OMP for sparse decomposition is to find the optimal atoms from the redundant dictionary that can best match the image signal. The calculation process is as follows.

First, select the atom ***g***_*γ*0_ that best matches the image signal ***f*** to be decomposed from the redundant dictionary, and meet the following conditions,

$$\left|\langle f,g_{\gamma 0}\rangle \right|=\underset{\gamma \in \Gamma }{\text{max}}\left|\langle f,g_{\gamma }\rangle \right|$$ (2)

Where, 〈•〉 represents the inner product. The image can be decomposed into two parts: the component on the best atom ***g***_*γ*0_ and the residual, namely,

$$f=\langle f,g_{\gamma 0}\rangle g_{\gamma 0}+R^{1}f$$ (3)

Where, *R*^1^***f*** is the residual error after matching the original image with the best atom. Continuously carry out the above decomposition process on the residual,

$$R^{k}f=\langle R^{k}f,g_{\gamma k}\rangle g_{\gamma k}+R^{k+1}f$$ (4)

Where, $\langle R^{k}f,g_{\gamma_{k}}\rangle$ represents the component of the image residual *R*^*k*^***f*** on the corresponding atom $g_{\gamma_{k}}$.

After *K* times decomposition, the image is decomposed into,

$$f=\sum_{k=0}^{K-1}\langle R^{k}f,g_{\gamma_{k}}\rangle g_{\gamma_{k}}+R^{K}f$$ (5)

Under the condition that the signal satisfies the limited length, the exponential decay ‖*R*^*k*^***f***‖ is becomes zero with the increase of *k*. A small number of atoms can represent the main components of the image, namely,

$$f\approx \sum_{k=0}^{K-1}\langle R^{k}f,g_{\gamma_{k}}\rangle g_{\gamma_{k}}$$ (6)

Where, *K* << *N*.

Assume the optimal atom set is ***Θ***_*K*_, the original signal could be represented by the *K* atoms, denoted as,

$$f=\Theta_{K}\theta$$ (7)

Where, ***θ*** is the sparse coefficient vector.

Utilize least square method to solve the sparse coefficient vector,

$$\hat{\theta }=\Theta_{K}^{+}f$$ (8)

The reconstructed signal could be expressed as,

$$\hat{f}=\Theta_{K}^{}\Theta_{K}^{+}f$$ (9)

The decomposition steps of OMP can be summarized in Algorithm 1.

Algorithm 1: OMP

Inputs: the original signal ***f***, the redundant dictionary ***D***, the atom number *K*.

Output: the reconstructed signal $\hat{f}$.

1: Initialization: set residual ***R***^0^***f*** = ***f***, optimal atom set ***Θ***_*K*_ = [], number of decompositions *k* = 1.

2: While *k* ≤ *K* do

3: Find the atom ***g***_*γk*_ that best matches the residuals from the redundant dictionary, which satisfies $g_{\gamma k}=\underset{\gamma \in \Gamma }{\text{max}}\left|\langle R^{k}f,g_{\gamma }\rangle \right|$.

4: Update the optimal atoms set with ***g***_*γk*_ to obtain ***Θ***_*k*_ = ***Θ***_*k*−1_ ⋃ ***g***_*γk*_.

5: Update the residual with $R^{k}f=f-\Theta_{k}\Theta_{k}^{+}f$, where $\Theta_{k}^{+}$ represents the pseudo-inverse of the matrix ***Θ***_*k*_.

6: Compute *k* = *k* + 1.

7: end

8: Compute the reconstructed signal $\hat{f}$ according to Eq (9).

### Sparse decomposition optimization model

Analyzing the sparse decomposition process of the OMP algorithm, we find that the selection of the optimal atom is completed by calculating the inner product of all the atoms in the redundant dictionary and the decomposition residuals. In order to improve the accuracy and efficiency of sparse decompositionprocess, this paper takes, 1) the maximum inner product of the matching atom and the image residual, and 2) the minimum correlation between the atoms, as the two optimization goal, to construct a multi-objective sparse decomposition optimization model.

According to Algorithm 1, after *k* − 1 sub-decomposition, the residual is *R*^*k*−1^***f***, and the optimal atom set is ***Θ***_*k*−1_, then in the *k*th decomposition process, the first objective function that needs to be optimized is the maximum inner product of matching atoms and residuals. The first objective function is computed as,

$$f_{1}\left(g_{\gamma }\right)=\underset{\gamma \in \Gamma }{\text{max}}\left|\langle R^{k\text{-}1}f,g_{\gamma }\rangle \right|$$ (10)

In order to ensure the orthogonality between atoms and make the decomposition process more accurate, the second objective function that needs to be optimized is the minimum correlation between atoms. The second objective function is computed as,

$$f_{2}\left(g_{\gamma }\right)=\underset{\gamma \in \Gamma }{\text{min}}\left|\langle \Theta_{k-1},g_{\gamma }\rangle \right|$$ (11)

In order to ensure the unity of the multi-objective solution process, taking the minimum of each objective function as the optimization objective. The multi-objective sparse decomposition optimization model is expressed as,

$$f=\left[\underset{\gamma \in \Gamma }{\text{min}}\frac{1}{\left|\langle R^{k\text{-}1}f,g_{\gamma }\rangle \right|},\underset{\gamma \in \Gamma }{\text{min}}\left|\langle \Theta_{k-1},g_{\gamma }\rangle \right|\right]$$ (12)

## Proposed NSGA-III-OMP algorithm

The basic idea of NSGA-III-OMP algorithm is: in the iterative process of OMP sparse decomposition, the NSGA-III evolution process is used to replace the original atom matching process. The main advantage of the algorithm NSGA-III is that, by defining a hyperplane and multiple reference points, it selects the individuals which can best adapt the environment, in order to maintain the diversity of the population and ensure that decision makers can find the optimal solution. Firstly, we describe the NSGA-III algorithm in detail, including the initilization, selection, the crossover and mutation operation. Following that, the flow chart and implementation of NSGA-III-OMP is at the last.

### NSGA-III algorithm

In view of the characteristics of the evolutionary algorithm to solve the multi-objective sparse decomposition model, there is no need to generate a Gabor dictionary in advance, and a real-valued encoding method is used to construct chromosomes, and each chromosome represents an atom. According to the generating formula of Gabor atom, the length of chromosome is *D* = 4, and its value is the vector represented by (*s*,*u*,*υ*,*ω*).

Suppose the population size is *M*, the maximum evolutionary generation is *G*_max_, and the number of objective functions is *P*. Genetic operator is the key to ensure the optimization of chromosome update, usually including selection, crossover and mutation [22, 23].

#### Initialization operation

The initial parent population $X_{0}^{\text{par}}$ is generated in a random manner. The *m*th element in $X_{0}^{\text{par}}$ is expressed in Eq (13),

$$X_{0}^{par}\left(n,m\right)=\frac{1}{\sqrt{s}}e^{-\pi {\left(\frac{n-u}{s}\right)}^{2}}\text{cos}\left(\upsilon n+\omega \right)$$ (13)

Where, (*s*,*u*,*υ*,*ω*) is the random number satisfying the range of specific parameter, *m* = 1,2,…,*M*.

#### Selection operation

The selection mechanism of the NSGA-III algorithm [24] is different from the traditional genetic algorithm. The specific steps include: non-dominant sorting, define the reference point, normalize objective function, association operation and filter operation.

Before the first selection operation, crossover and mutation operations are performed on the initial parent population $X_{0}^{\text{par}}$ to generate offspring population $X_{0}^{\text{spr}}$. Merge the parent population $X_{0}^{\text{par}}$ and the offspring population $X_{0}^{\text{spr}}$ to form a composite population $X_{0}^{\text{com}}=X_{0}^{\text{par}}⋃X_{0}^{\text{spr}}$.

*1) Non-dominant sorting*. Compute the fitness and take the top *M* members from the composite population $X_{0}^{\text{com}}$. The individuals of the composite population $X_{0}^{\text{com}}$ are divided into *L* layers according to the non-dominated rules [19]. The first *L* − 1 layers of individuals is denoted as ***X***_sel_ and the number is |***X***_sel_|. If |***X***_sel_| = *M*, the next parent population is started with $X_{G+1}^{\text{par}}=X_{\text{sel}}$, else we need to select other *M* − |***X***_sel_| members from the *L*th layer.

*2) Define the reference point*. We employ an efficient technique that spaces points on a normalization hyperplane with an intercept of one on each axis and is equally inclined to all objective axes. The number of objective functions is *P*, the length of chromosome is *D*, then the number of reference points is $J=C_{P+D-1}^{D}$, *j* = 1,2,…,*J*, and the reference points can be set on the normalized hyperplane of dimension *P* − 1. We believe that the optimal solutions are widely scattered across the complete normalized hyper-plane.

*3) Normalize objective function*. Calculate the ideal point in population ***X***_sel_ by recognizing the minimum value $b_{p}^{\text{min}}$ for each objective function *p* = 1,2,…,*P*, and build the ideal point $\left\{b_{1}^{\text{min}},b_{2}^{\text{min}},\ldots ,b_{P}^{\text{min}}\right\}$, then the translated objective is computed by Eq (15),

$$b_{p}^{\text{min}}=\text{min}\;f_{p}\left(z\right),z\in X_{\text{sel}}$$ (14)

$$f_{p}^{\prime }\left(z\right)=f_{p}\left(z\right)-b_{p}^{\text{min}}$$ (15)

The intercept *a*_*P*_ of the *p*-th objective axis and the linear hyper-plane can then be computed according to the reference [19], and the normalized objective functions can be represented as,

$$f_{p}^{\text{n}}\left(z\right)=\frac{f_{p}^{\prime }\left(z\right)}{a_{p}-b_{p}^{\text{min}}}=\frac{f_{p}\left(z\right)-b_{p}^{\text{min}}}{a_{p}-b_{p}^{\text{min}}},\;for\;p=1,2,\ldots ,P$$ (16)

Assume the structured reference points is *H*^s^, we utilize Eq (16) to map each reference point onto the normalized hyperplane, and save the reference points in *H*^n^.

*4) Association operation*. Connect the reference point and the origin of hyperplane to form a reference line, calculate the perpendicular distance from all individuals in ***X***_sel_ to the reference lines. Select the nearest reference line, and associate the individual with the corresponding reference point. Through association operation, we associate individuals with reference points. It may appear that a reference point is associated with one or more population individuals, or some reference points are not associated with any population individual. At this point, we need to perform filter operation.

*5) Filter operation*. Determine the number *ρ*_*j*_ of first *l* − 1 layer individuals associated with each reference point *j*, identify the reference points set *J*_min_ = {*j*:argmin_*j*_ *ρ*_*j*_} having minimum *ρ*_*j*_. Randomly select a reference point from *j*_1_, if $\rho_{j_{1}}=0$ and *j*_1_ is associated with multiple individuals in *l*th layer, then select the individual closest to the reference line *j*_1_*O* to enter the next generation. If $\rho_{j_{1}}=0$ and *j*_1_ is not associated with multiple individuals in *l*th layer, then randomly select an individual to enter the next generation. Repeatedly select reference points from *J*_min_, until the individuals are supplemented [19] and the parent population $X_{G+1}^{\text{par}}$ operation is completed.

#### Crossover and mutation operation

Since we have already performed an elitist selection operator on the parent population, the population diversity could be maintained. Therefore, we apply usual crossover and mutation operators to create the offspring population $X_{G+1}^{\text{spr}}$.

According to the crossover probability, it is judged whether the individual has crossover and the gene position where the crossover occurs. The analog binary crossover operator is used to generate the progeny population. According to the mutation probability, determine whether the individual has mutation and determine the gene location where the mutation occurred, and adopt the basic mutation method to determine the individual value after mutation.

#### NSGA-III process

After continous iteration in NSGA-III evolution process, we obtain the optimal offspring solution set $X_{G_{\text{max}}}^{\text{spr}}$. Compute the contribution degree of the individual objective function value in the total objective function value, the individual ***X***_optimal_(optimal = 1,2,…,*M*) with the smallest value of *f*_optimal_ is selected as the optimal atom. The smallest value of *f*_optimal_ is computed as,

$$f_{optimal}=\sum_{p=1}^{P}\frac{\left(f_{p}\left(X_{optimal}\right)-{\left(f_{p}\left(X_{m}\right)\right)}_{\text{min}}\right)}{{\left(f_{p}\left(X_{m}\right)\right)}_{\text{min}}}$$ (17)

Where, $X_{m}^{}\left(m=1,2,\ldots ,M\right)\in X_{G_{\text{max}}}^{\text{spr}}$.

Combined with the description of the above operators, the implementation steps of NSGA-III is summarized in Algorithm 2.

**Algorithm 2: NSGA-III**

**Inputs**: the evolution generation *G*_max_, the initial parent population $X_{0}^{\text{par}}$, the objective function *f*_*p*_, *p* = 1,2,…,*P*, the structured reference points *H*^s^.

**Output**: the optimal solution ***X***_optimal_.

**1**: Perform crossover and mutation operations to generate the initial offspring population $X_{0}^{\text{spr}}$, set iteration *G* = 0.

**2**: **While** *G* ≤ *G*_max_ **do**

**3**: ***X***_sel_ = *ϕ*, *l* = 1

**4**: $X_{G}^{\text{com}}=X_{G}^{\text{par}}\cup X_{G}^{\text{spr}}$

**5**: Select one individual ***X***_*l*_ from $X_{G}^{\text{com}}$ according non-dominant sorting.

**6**: **repeat**

**7**:  ***X***_sel_ = ***X***_sel_ ⋃ ***X***_*l*_

**8**:  Compute *l* = *l* + 1

**9**: **until** *l* = *L* − 1

**10**: **if** |***X***_sel_| = *M* **then**

**11**:  $X_{G+1}^{\text{par}}=X_{\text{sel}}$,

   break;

**12**: **else**

**13**:  $X_{G+1}^{\text{par}}=X_{\text{sel}}$

**14**:  Points to be choosen from *L*th layer: *M* − |***X***_sel_|

**15**:   Normalize the objective functions using Eq (16) and save the reference points *H*^n^.

**16**:  Associate each member of ***X***_sel_ with a reference point.

**17**:  Excute the filter operator to obtain the parent population $X_{G+1}^{\text{par}}$.

**18**: **endif**

**19**: Perform crossover and mutation operations to generate the offspring population $X_{G+1}^{\text{spr}}$.

**20**: Compute *G* = *G* + 1

**21**: **endWhile**

**22**: Find the optimal solution ***X***_optimal_ using Eq (17) from $X_{G_{\text{max}}}^{\text{spr}}$.

### Implementation of NSGA-III-OMP

We use the NSGA-III algorithm to replace the atomic matching process in OMP, and propose the NSGA-III-OMP algorithm. The implementation of NSGA-III-OMP is summarized in Algorithm 3.

**Algorithm 3: NSGA-III-OMP**

**Inputs**: the original signal ***f***, the atom number *K*, the parameters of NSGA-III (including the population size *M*, the maximum evolution generation *G*_max_).

**Output**: the reconstructed signal $\hat{f}$.

**1**: Initialize residual *R*^0^***f*** = ***f***, the optimal atoms set ***Θ***_*k*_ = [], number of decompositions *k* = 1.

**2**: **While** *k* ≤ *K* **do**

**3**: According to Algorithm 2: NSGA-III, with the parameters *M* and *G*_max_, the optimal individual ***X***_optimal_ is obtained as the optimal atom ***g***_*k*_.

**3**: Update the optimal atoms set with ***g***_*γk*_ to obtain ***Θ***_*k*_ = ***Θ***_*k*−1_ ⋃ ***g***_*γk*_.

**4**: Update residual with $R^{k}f=f-\Theta_{k}\Theta_{k}^{+}f$, where $\Theta_{k}^{+}$ represents the pseudo-inverse of the matrix ***Θ***_*k*_.

**5**: Compute *k* = *k* + 1.

**6**: **end**

**7**: Compute reconstructed signal $\hat{f}$ according to Eq (9).

## Experimental datasets and evaluation metrices

### Hyperspectral datasets

**Fig 1:**
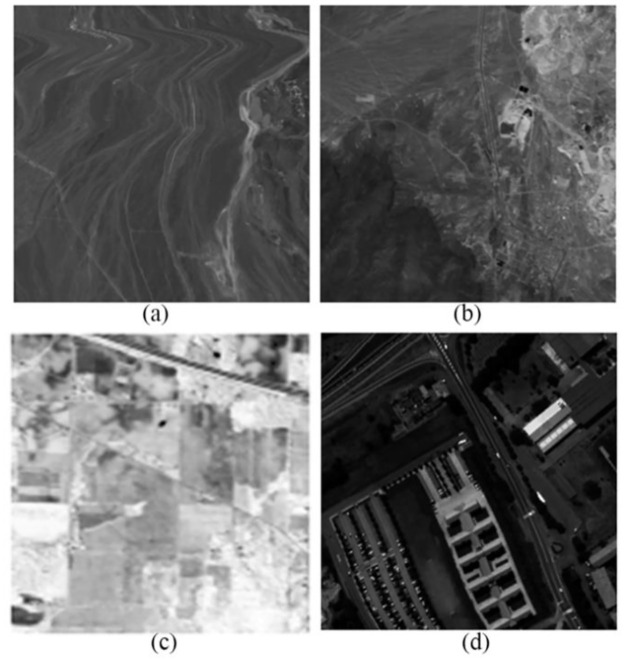
Original 50th images of four datasets. (a) Cuprite1. (b) Cuprite2. (c) Indian Pines. (d) Pavia University.

Four hyperspectral datasets [25, 26] with diverse signatures are included in the investigations, allowing for a complete quantitative and quantitative comparative evaluation of the proposed scheme. The datasets are namely Cuprite1, Cuprite2, Indian Pines collected by AVIRIS and Pavia University collected by ROSIS. The water absorption and noisy bands in the original data set have been removed, and the image has been spatially cropped to the block size *B* = 16 for computation convenience. The basic conditions of the 4 sets of data are shown in S1 Table. The original image of the 50th band of hyperspectral data is shown in Fig 1.

### Evaluation metrices

The performance of the algorithm is evaluated using the average peak signal-to-noise ratio (PSNR) [27], average structural similarity (SSIM) [28], average vector-based SNR [29] and average vector-based spectral-angle distortion (SAD) [29], running time and reconstructed image. The running software and hardware environment of the experiment is: AMD quad-core CPU, 3.80 GHz, 16 G memory, Matlab2012b.

In the next discussions, we use ***F*** = [***F***_1_, ***F***_2_, …***F***_*l*_,…***F***_*L*_] ∈ *R*^*N*×*L*^ to represent the hyperspectral images, *N* is the pixels of each band image and *L* is the number of bands, ***F***_*l*_ is the vector representation of one band image, ***F***[*n*] is the vector representation of one pixel.

The band-based PSNR measured in dB is defined as,

$$\text{PSNR}\left(F_{l},{\hat{F}}_{l}\right)=20{\;\text{log}}_{10}\frac{\text{max}\left(F_{l}\right)}{\sqrt{\text{MSE}\left(F_{l},{\hat{F}}_{l}\right)}}$$ (18)

Where, ***F***_*l*_ and ${\hat{F}}_{l}$ are the original and reconstructed band images, max(***F***_*l*_) is the peak value of ***F***_*l*_, $\text{MSE}\left(F_{l},{\hat{F}}_{l}\right)$ is the mean squared error [27] and is computed as,

$$\text{MSE}\left(F_{l},{\hat{F}}_{l}\right)=\frac{1}{N}{\left\|F_{l}-{\hat{F}}_{l}\right\|}_{2}^{2}$$ (19)

The band-based SSIM between ***F***_*l*_ and ${\hat{F}}_{l}$ is computed as,

$$\text{SSIM}\left(F_{l},{\hat{F}}_{l}\right)=\frac{\left(2\mu_{1}\mu_{2}+C_{1}\right)\left(2\sigma_{12}+C_{2}\right)}{\left(\mu_{1}^{2}+\mu_{2}^{2}+C_{1}\right)\left(\sigma_{1}^{2}+\sigma_{2}^{2}+C_{2}\right)}$$ (20)

Where, *μ*_1_ and *μ*_2_ are the mean values of ***F***_*l*_ and ${\hat{F}}_{l}$, *σ*_1_ and *σ*_2_ are the standard deviation values of ***F***_*l*_ and ${\hat{F}}_{l}$, *σ*_12_ represents the correlation coefficient between ***F***_*l*_ and ${\hat{F}}_{l}$, *C*_1_ and *C*_2_ are constants related to the dynamic range of the pixel values. The details for these parameters can refer to [28].

The average PSNR and average SSIM are calculated by averaging the band-based PSNR and band-based SSIM over all bands of the hyperspectral dataset.

Vector-based SNR measured in dB is defined as,

$$\text{SNR}\left(F\left[n\right],\hat{F}\left[n\right]\right)=10{\;\text{log}}_{10}\frac{\text{var}\left(F\left[n\right]\right)}{\text{MSE}\left(F\left[n\right],\hat{F}\left[n\right]\right)}$$ (21)

Where, ***F***[*n*] and $\hat{F}\left[n\right]$ shows the original and rebuilt spectral vectors, var(***F***[*n*]) shows the variance of ***F***[*n*], and $\text{MSE}\left(F\left[n\right],\hat{F}\left[n\right]\right)$ is the mean squared error, defined as,

$$\text{MSE}\left(F\left[n\right],\hat{F}\left[n\right]\right)=\frac{1}{L}{\left\|F\left[n\right]-\hat{F}\left[n\right]\right\|}_{2}^{2}$$ (22)

The vector-based SAD measured in degree is presented as,

$$\text{SAD}\left(F\left[n\right],\hat{F}\left[n\right]\right)=∠\left(F\left[n\right]-\hat{F}\left[n\right]\right)$$ (23)

The average SNR and average SAD are calculated by averaging the vector-based SNR and vector-based SAD over all spectral vectors in the hyperspectral dataset.

## Parameter selection

The proposed algorithm NSGA-III-OMP needs to set the number of populations, the maximum evolutionary algebra and the number of decompositions (ie, the optimal number of atoms), and the effects of these parameters on the performance of the algorithm are studied separately.

### Evolution parameter

Firstly, the algorithm NSGA-III-OMP is used to sparsely decompose the 40th band image of the 4 groups of hyperspectral data, and the influence of the maximum evolutionary generation, population size and decomposition times on the performance of the algorithm is analyzed. The variation range of population size *M* is 5~50, the interval is 5, the variation range of the maximum evolutionary generation *G*_max_ is 5~50, the interval is 5, the variation range of the decomposition number *K* is 20~100, and the interval is 20.

**Fig 2:**
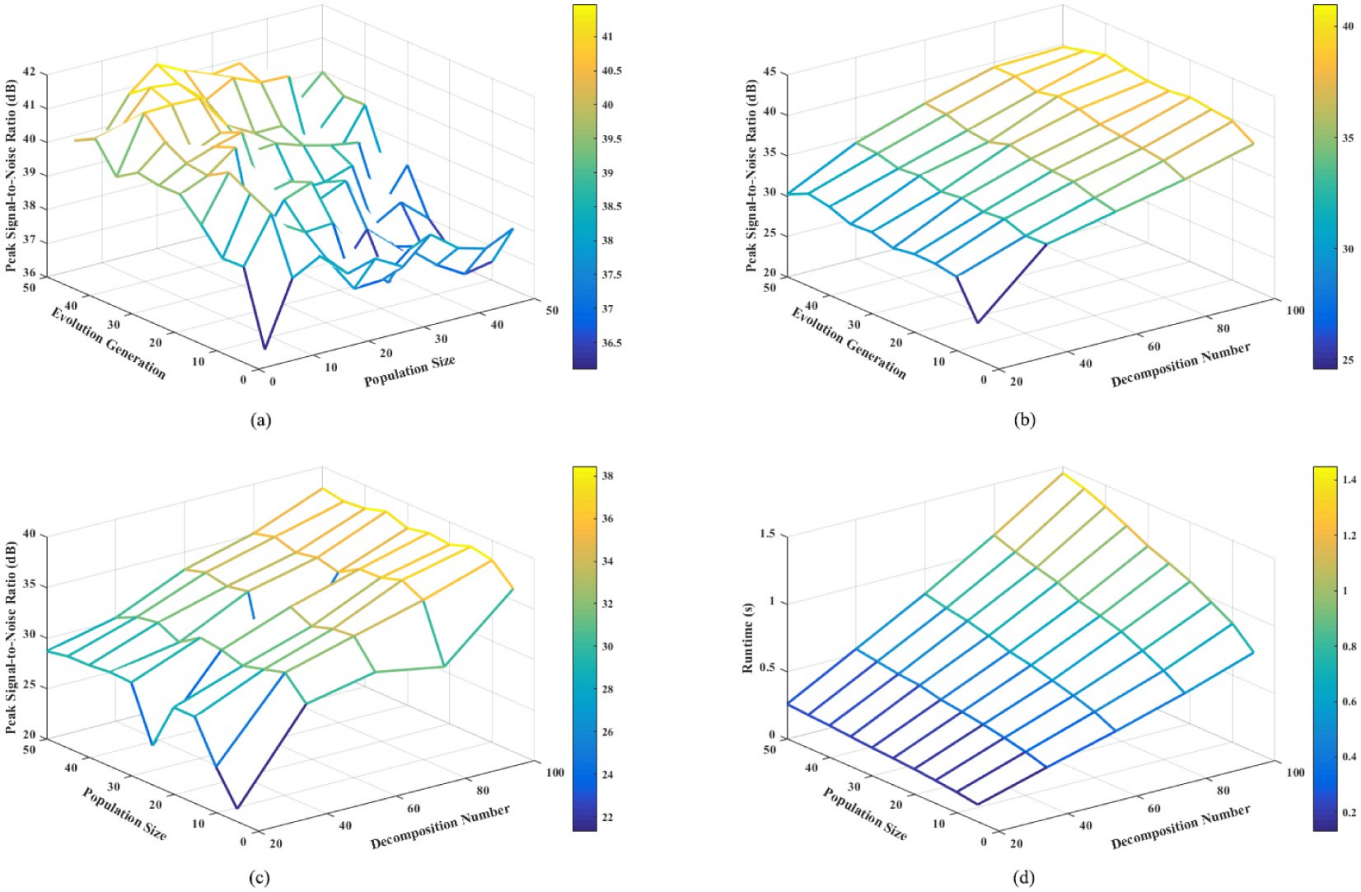
Influence of evolution parameters on NSGA-III-OMP. (a) Influence of maximum evolution generation and population size on reconstructed PSNR, when the decomposition number is 100. (b) Influence of maximum evolution generation and decomposition number on reconstructed PSNR, when the population size is 10. (c) Influence of population size and decomposition number on reconstructed PSNR, when the maximum evolution generation is 5. (d) Influence of maximum evolution generation and population size on reconstructed time, when the decomposition number is 100.

Fig 2 shows the variation of the average PSNR of the reconstructed image of Cuprite2 with the parameters. When the number of decompositions is 100, the average reconstructed PSNR varies with the maximum evolutionary generation and population size is shown in Fig 2(a). Under the same maximum evolutionary generation, the PSNR has a small oscillation with the increase of the number of populations. While under the small population number, with the increase of the evolutionary generation, the PSNR has a slow increase, but the overall change is not large.

When the population size is 10, the influence of the maximum evolutionary generation and decomposition times on the reconstructed PSNR is shown in Fig 2(b). When the maximum evolutionary generation is 5, the influence of population size and decomposition times on reconstruction performance is shown in Fig 2(c). From Fig 2(b) and 2(c), it can be clearly found that the influence of the number of decompositions on the reconstructed PSNR is much greater than the influence of the maximum evolutionary generation and the number of populations.

Fig 2(d) shows the influence of the maximum evolutionary generation and population size on the reconstruction time when the decomposition number is 100. It is found that the maximum evolutionary generation has a greater impact on the reconstruction efficiency than the population size. The experimental results of the other three groups of hyperspectral images are similar to Cuprite2, considering the reconstruction accuracy and computational complexity, the number of selected population size in NSGA-III-OMP algorithm is *M* = 10, and the maximum evolutionary generation is *G*_max_ = 5.

### Decomposition number

**Fig 3:**
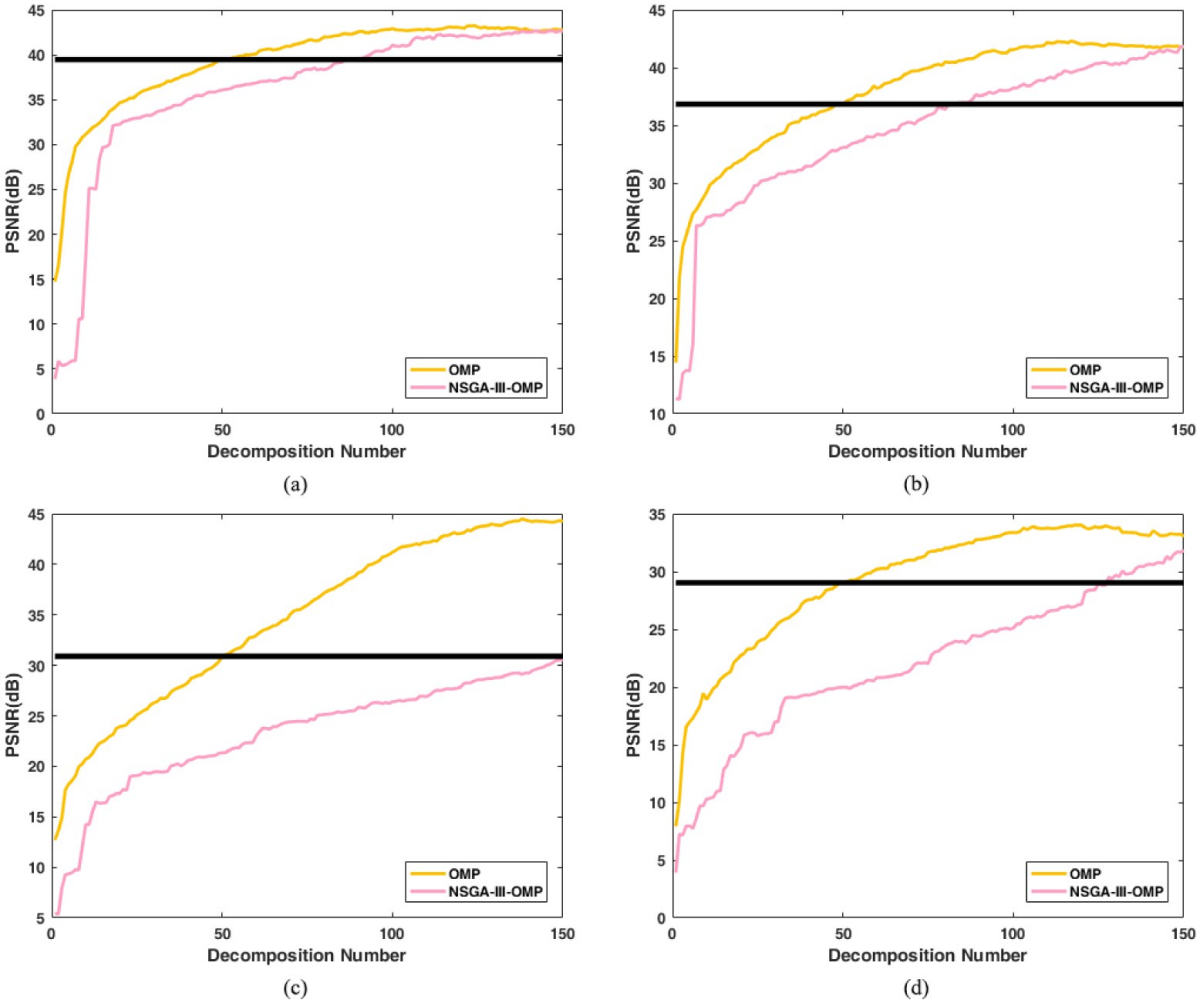
Reconstructed PSNR vs decomposition number of algorithm OMP and algorithm NSGA-III-OMP. (a) Reconstructed PSNR vs decomposition number for Cuprite1. (b) Reconstructed PSNR vs decomposition number for Cuprite2. (c) Re-constructed PSNR vs decomposition number for Indian Pines. (d) Reconstructed PSNR vs decomposition number for Pavia University. Note: Thick line is the accuracy of the reconstructed image obtained by the 50 best atoms using OMP.

The NSGA-III-OMP algorithm and the OMP algorithm are used to sparsely decompose the 40th band image of the hyperspectral data sets, and the algorithm is set to terminate when the number of decompositions reaches 150. Fig 3 shows the changes of the reconstructed PSNR obtained by the two algorithms. The thick line is the accuracy of the reconstructed image obtained by the 50 best atoms using OMP.

The experimental results show that the NSGA-III-OMP algorithm cannot achieve the reconstruction accuracy of the OMP algorithm with only 50 atoms. This is because: in each decomposition process of the OMP algorithm, it can find the best matching atoms from the redundant dictionary to guarante the performance. Due to the randomness, the atom searched by NSGA algorithm may not be the atom that best matches the residual. Therefore, more atoms need to be found to fully characterize the original image characteristics and achieve the reconstruction accuracy of the OMP algorithm.

If the PSNR of the reconstructed image obtained by the OMP algorithm using 50 atoms is used as the standard, Cuprite1 and Cuprite2 need about 100 atoms to achieve this standard, while Indian Pines and Pavia University need about 150 atoms to achieve the accuracy of the OMP algorithm. Accordingly, the maximum decomposition number of the OMP algorithm is set to *K* = 50. For the 4 sets of hyperspectral data, the decomposition number for the algorithm NSGA-III-OMP is set to *K* = [100,100,150,150].

## Results and analysis

Three decomposition techniques are compared in order to better understand the possibilities of the proposed NSGA-III-OMP. The first is the OMP algorithm, described in Algorithm 1. This approach searches the best atoms by traversing all the atoms in the redundant dictionary. The second is the particle swarm optimization based on OMP, denoted as PSO-OMP algorithm, described in literature [30]. The core idea of this algorithm is within the framework of OMP, achieving the best atoms by evolution of particle swarms. The third one is our proposed algorithm, presented in Algorithm 3. This algorithm is within the framework of OMP, exploring the genetic evolution to search the best atoms. Compared with PSO-OMP, except for the differences in evolutionary principles, our NSGA-III-OMP algorithm also optimizes a multi-objective model shown in subsection 2.3, while PSO-OMP solves a single objective model. In addition, the selection operation adopts a non-dominated sorting to maintain the diversity of population and ensure the accuracy of the optimal solution.

The decomposition number of OMP algorithm is *K* = 50. With reference [30], the population size of PSO-OMP algorithm is 10, the maximum evolutionary generation is 5, and the decomposition number is *K* = [100,100,150,150] for four datasets, respectively. The parameters of NSGA-III-OMP algorithm are: the population size is *M* = 10, the maximum evolution generation is *G*_max_ = 5, and the decomposition number is *K* = [100,100,150,150] for four datasets.

### Reconstruction performance comparison

**Fig 4:**
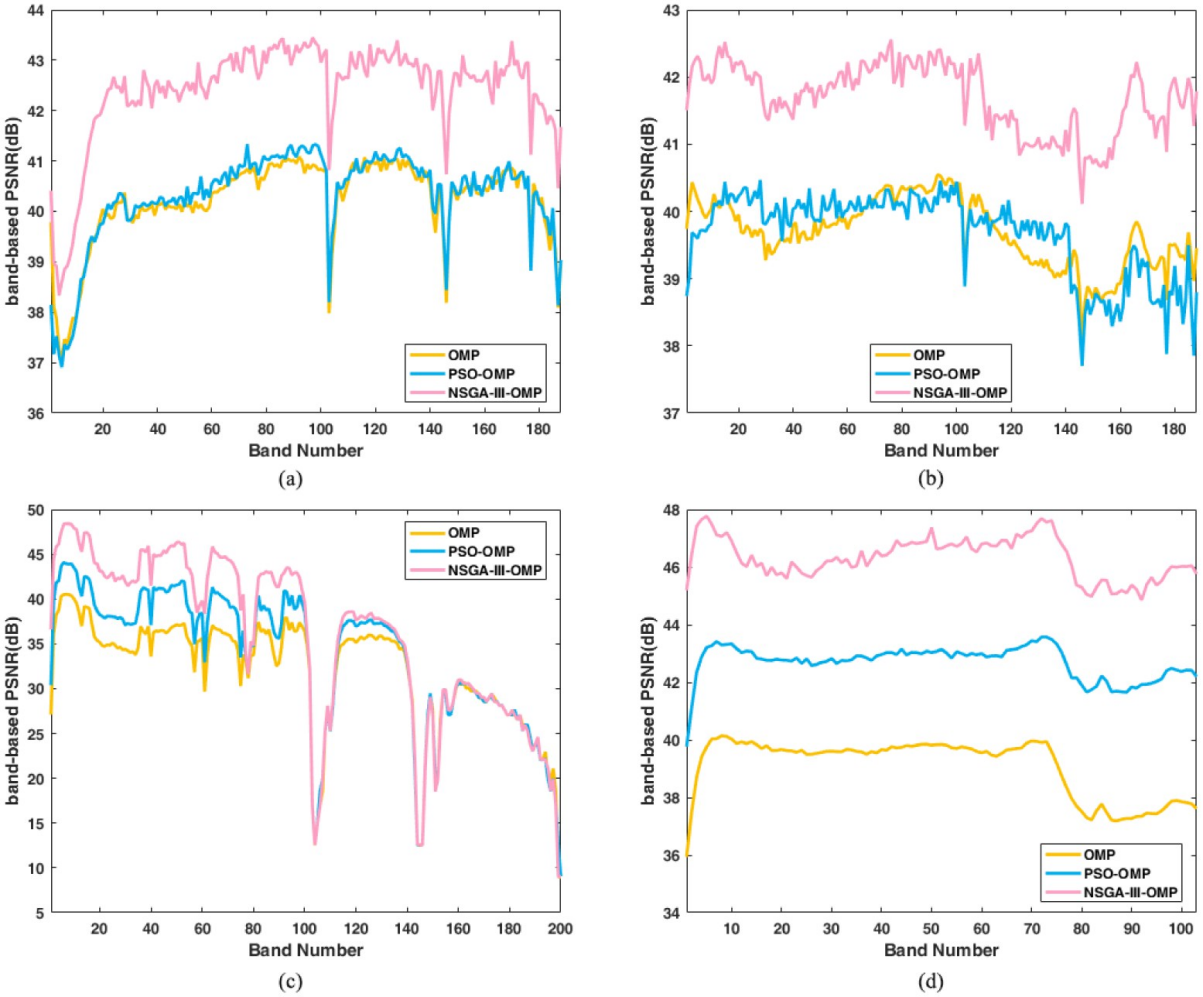
Reconstructed band-based PSNR vs band number using OMP, PSO-OMP and NSGA-III-OMP. (a) Reconstructed PSNR for Cuprite 1. (b) Reconstructed PSNR for Cuprite 2. (c) Reconstructed PSNR for Indian Pines. (d) Reconstructed PSNR for Pavia University.

The band-based PSNR of four datasets is shown in Fig 4. Seen from the figure, the results of PSO-OMP are almost the same as OMP for Cuprite1 and Cuprite2, shown in Fig 4(a) and 4(b). We can clearly find that the proposed NSGA-III-OMP has obvious advantage over PSO-OMP and OMP. In regard to Indian Pines in Fig 4(c), we discover that for first half bands (maybe 1–100 bands), the NSGA-III-OMP is better than PSO-OMP, and OMP is worst result. Whereas, in the last bands (maybe 101–200 bands), the three algorithms obtain the very consistent accuracy. For the dataset Pavia University, we can clearly see the advantages of evolution algorithms, especially our NSGA-III-OMP has absolute advantage over the other two methods. The average PSNR listed in Table 1 can get the same conclusion as in Fig 4. The average PSNR are computed by the band-based PSNR of four datasets with three methods (see S1 Dataset). These results shows that our algorithm can better find the optimal atoms by solving a multi-objective model using non-dominated sorting selection operation.

**Table 1:** Average PSNR obtained by using OMP, PSO-OMP and NSGA-III-OMP.

Algorithm	Average PSNR/dB			
	Cuprite1	Cuprite2	Indian Pines	Pavia University
**OMP**	40.21	39.69	31.89	39.07
**PSO-OMP**	40.30	39.66	33.78	42.69
**NSGA-III-OMP**	42.41	41.70	35.89	46.32

**Fig 5:**
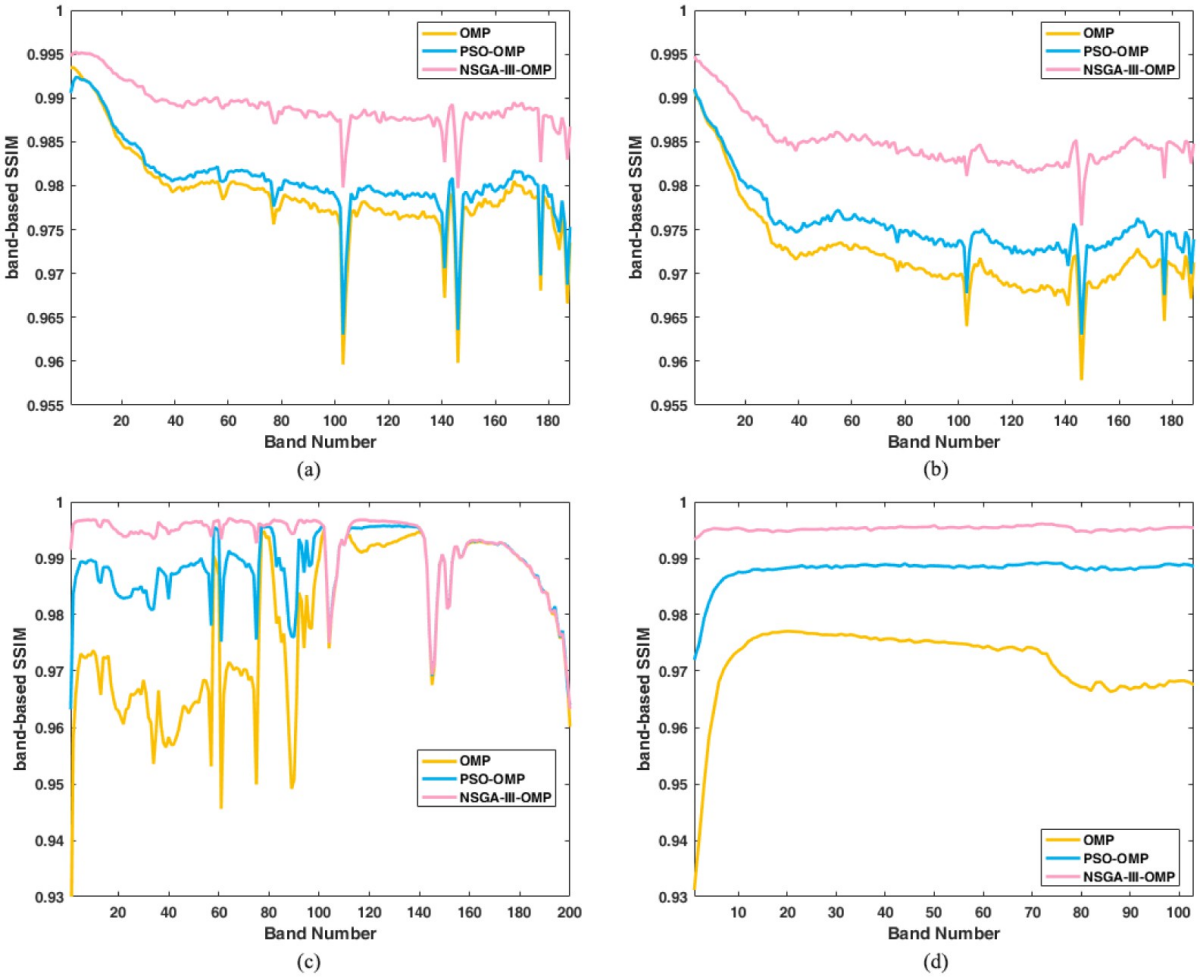
Reconstructed band-based SSIM vs band number using OMP, PSO-OMP and NSGA-III-OMP. (a) Reconstructed SSIM for Cuprite 1. (b) Reconstructed SSIM for Cuprite 2. (c) Reconstructed SSIM for Indian Pines. (d) Reconstructed SSIM for Pa-via University.

The band-based SSIM of four datasets is shown in Fig 5. For Cuprite1 and Cuprite2 images, the SSIM of PSO-OMP is higher than OMP, which is different from the PSNR comparison in Fig 4(a) and 4(b). Especially, the results of our proposed algorithm NSGA-III-OMP are superior to the other two methods. The average SSIM are presented in Table 2. The average SSIM are computed by the band-based SSIM of four datasets with three methods (see S2 Dataset). This shows that our algorithm can recover the spatial structural features of the original image, which is a compelling proof of the proposed algorithm’s usefulness.

**Table 2:** Average SSIM obtained by using OMP, PSO-OMP and NSGA-III-OMP.

Algorithm	Average SSIM			
	Cuprite1	Cuprite2	Indian Pines	Pavia University
**OMP**	0.9792	0.9722	0.9787	0.9717
**PSO-OMP**	0.9807	0.9756	0.9885	0.9879
**NSGA-III-OMP**	0.9890	0.9848	0.9928	0.9953

**Fig 6:**
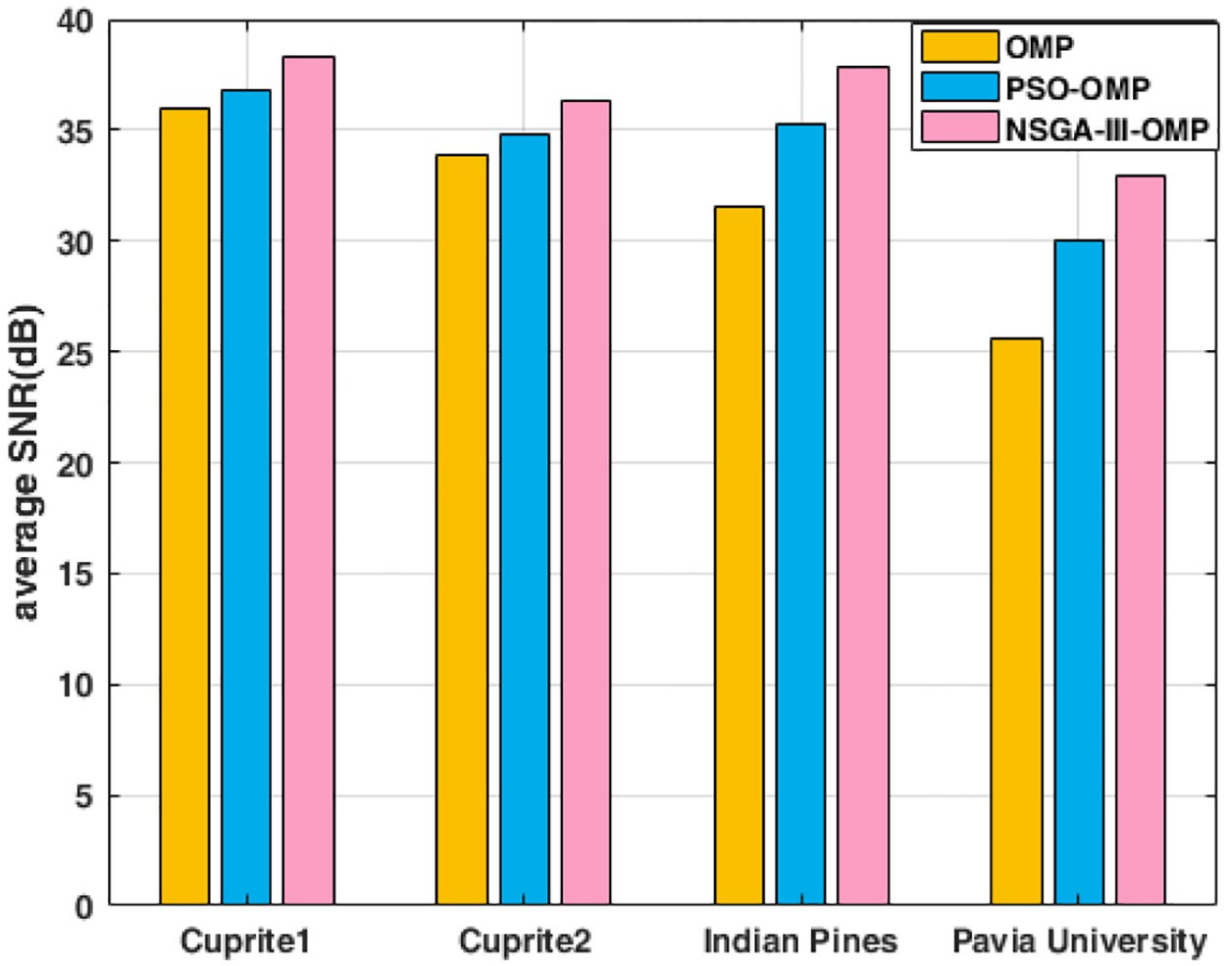
Reconstructed average SNR using OMP, PSO-OMP and NSGA-III-OMP for four datasets.

**Fig 7:**
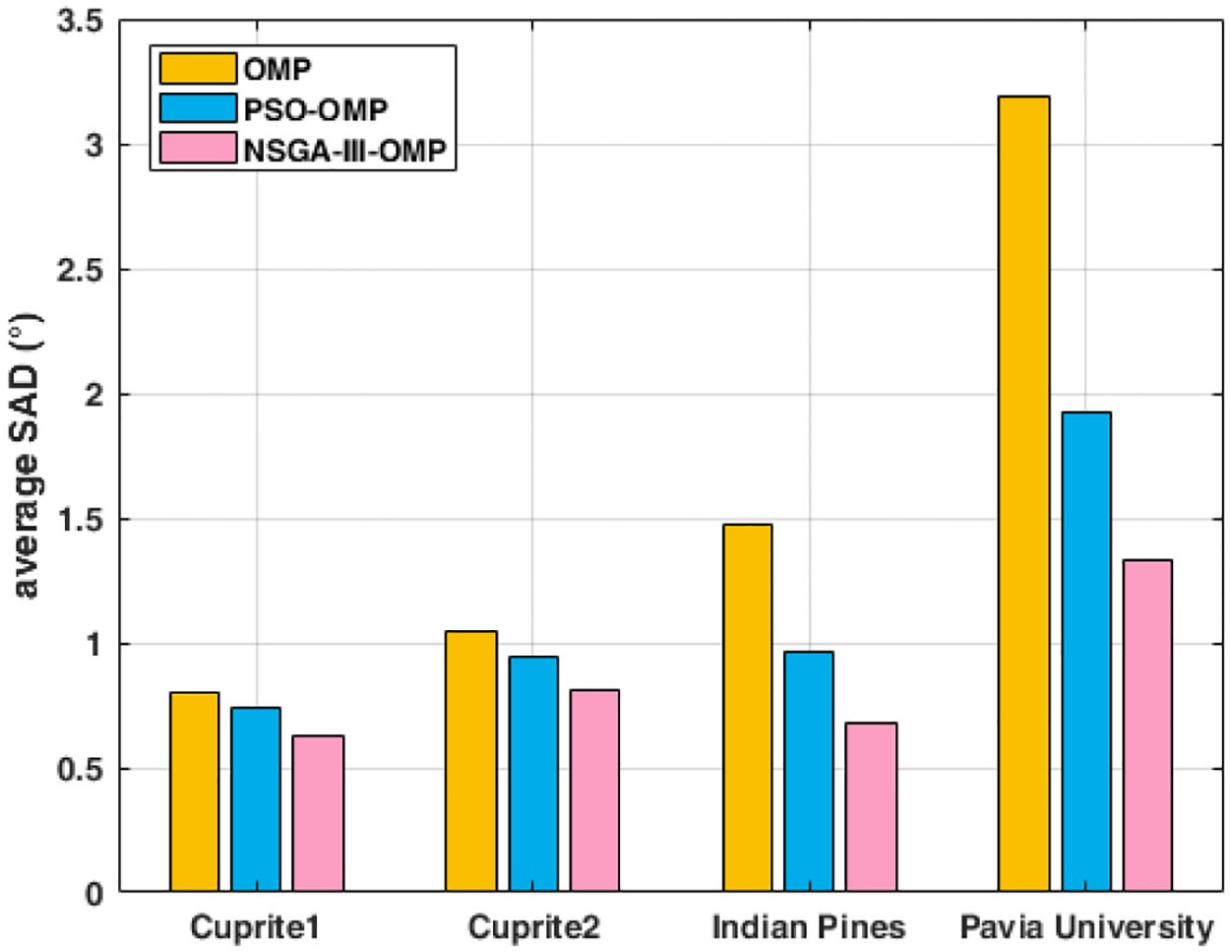
Reconstructed average SAD using OMP, PSO-OMP and NSGA-III-OMP for four datasets.

The average SNR and average SAD are shown in Fig 6 and Fig 7. Seen from Fig 6, our NSGA-III-OMP has the highest SNR among the three algorithms, which illustrates our algorithm can recover the spectral vestors accurately. The results in Fig 7 further explained the effectiveness of proposed NSGA-III-OMP, because the spectal angle distortion between the original spectral vector and the reconstructed spectral vector is the smallest. The average SNR are computed by the vector-based SNR of four datasets with three methods (see S3 Dataset). The average SAD are computed by the vector-based SAD of four datasets with three methods (see S4 Dataset).

**Fig 8:**
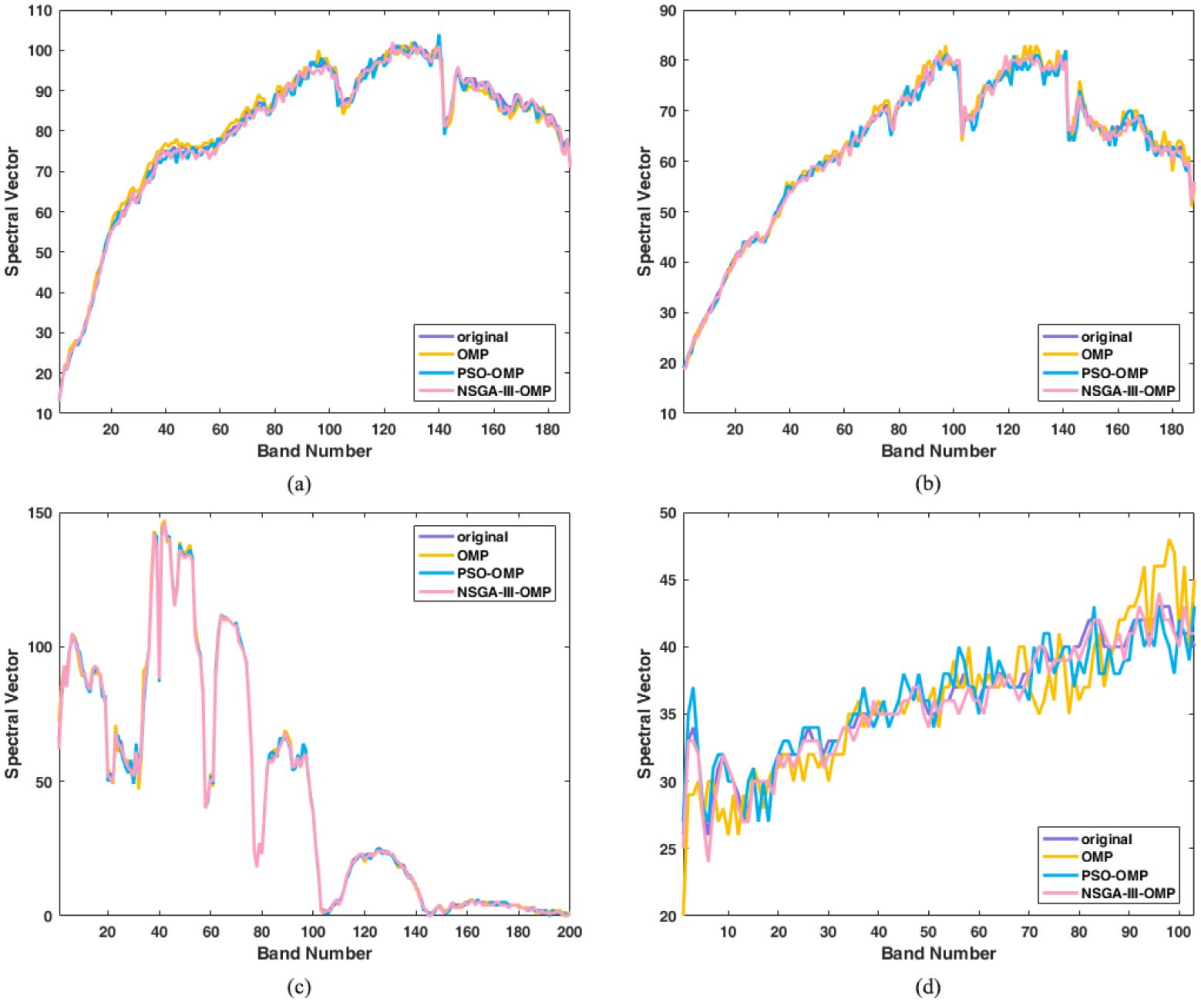
Comparison between reconstructed spectral vectors and original sepctral vector. (a) Reconstructed spectral vectors for Cuprite 1. (b) Reconstructed spectral vectors for Cuprite 2. (c) Reconstructed spectral vectors for Indian Pines. (d) Recon-structed spectral vectors for Pavia University.

The comparisons between the original spectral vector and the reconstructed spectral vectors are shown in Fig 8. In this figure, without loss of generality, we choose one spectral vector randomly from the datasets as a representive for comparison. The reconstructed spectral vectors are very consistent with the original spectral vectors, which is an addition proof of the algorithm’s avalibility.

**Fig 9:**
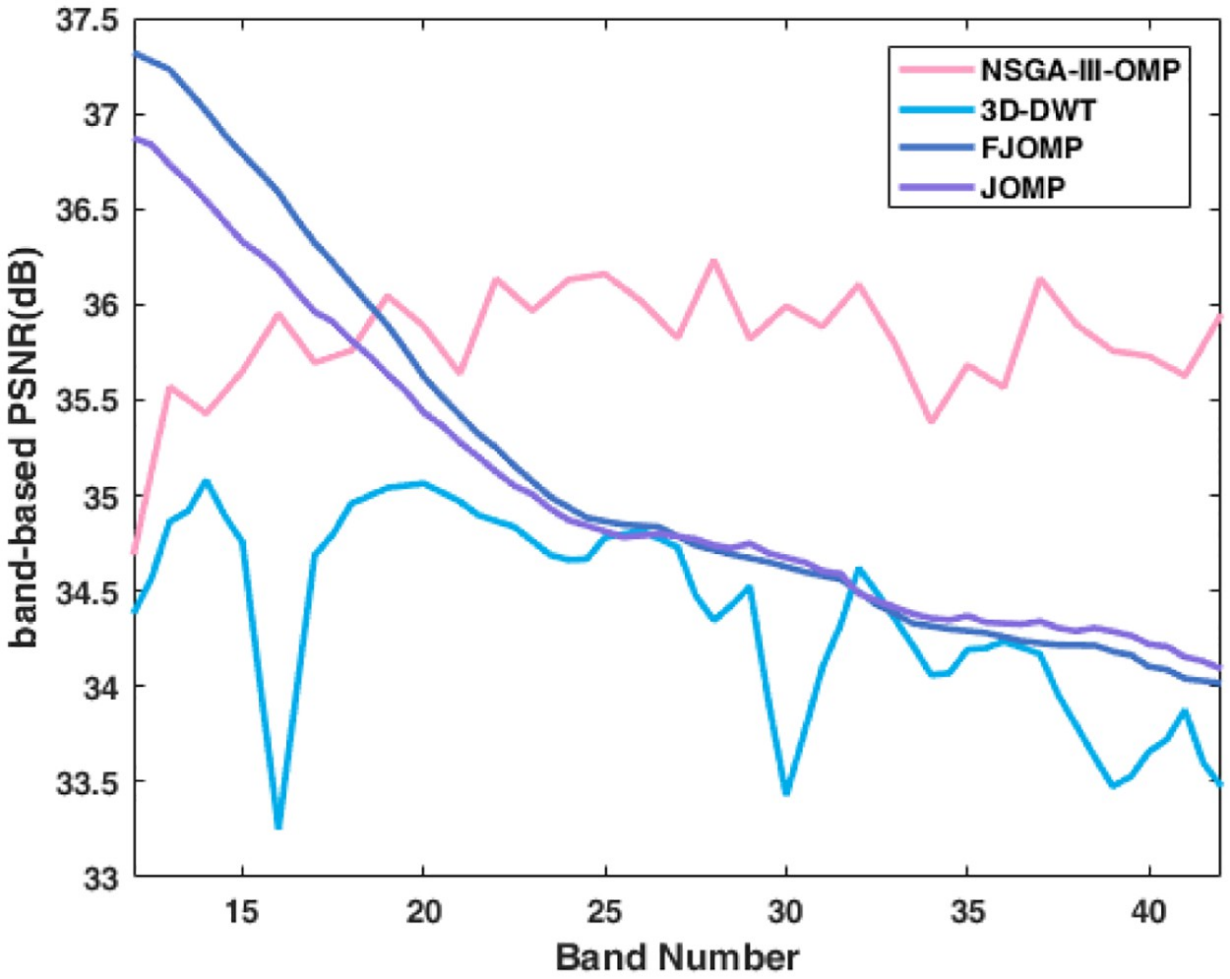
Comparison between our proposed NSGA-III-OMP and other three methods.

The following comparison with several other state-of-the-art algorithms is presented to further showcase the algorithm’s performance. The algorithm 3D-DWT [31] uses three dimension wavelet transform for the sparse decomposition of hyperspectral images. The JOMP [31] computes the common support by sparse coding the vector consisting of patches of all bands, leading to high complexity. The FJOMP [32] finds the common support by sparse coding the training band, improving the computational efficiency. Note that, the Cuptite1 image used in this compariosn is cut from the upper corner to be subimage of size 512×512 pixels for keeping in line with the literatures. Fig 9 shows the Cuprite1’s results in comparison to the algorithms 3D-DWT, JOMP and FJOMP employing band-based PSNR. The bands in Fig 9 are 12~42.

The average PSNR of 3D-DWT, JOMP, FJOMP, NSGA-III-OMP is 23.53 dB, 24.67 dB, 25.11 dB, 35.81 dB, respectively. Seen from the results, the PSNR of 3D-DWT is the worst, illustrating that for hyperspectral images with complex features, the sparse representation capabilities of orthogonal basis is not enough. For the first 12~20 bands, the PSNR of JOMP and FJOMP are higher than NSGA-III-OMP, while the two algorithms dropped significantly with the band number increasing. In contrast to our algorithm, the performance remains stable and obtains the highest average PSNR. Although the comparison is not detailed enough, the results can still reflect the effectiveness of our proposed algorithm.

In conclusion, numerous comparisons between the proposed NSGA-III-OMP and some state-of-the-art algorithms have demonstrated the reliability of the algorithm. In comparison with the OMP and PSO-OMP algorithms, the results and analysis fully indicate that it is feasible to use NSGA-III to solve the constructed multi-objective sparse decomposition optimization model. The optimal atoms searched by NSGA-III not only can represent the spatial features of the band images, but also describe the spectral features of spectral vectors. These results are able to prove the decomposition accuracy of NSGA-III-OMP.

### Reconstruction efficiency comparison

**Fig 10:**
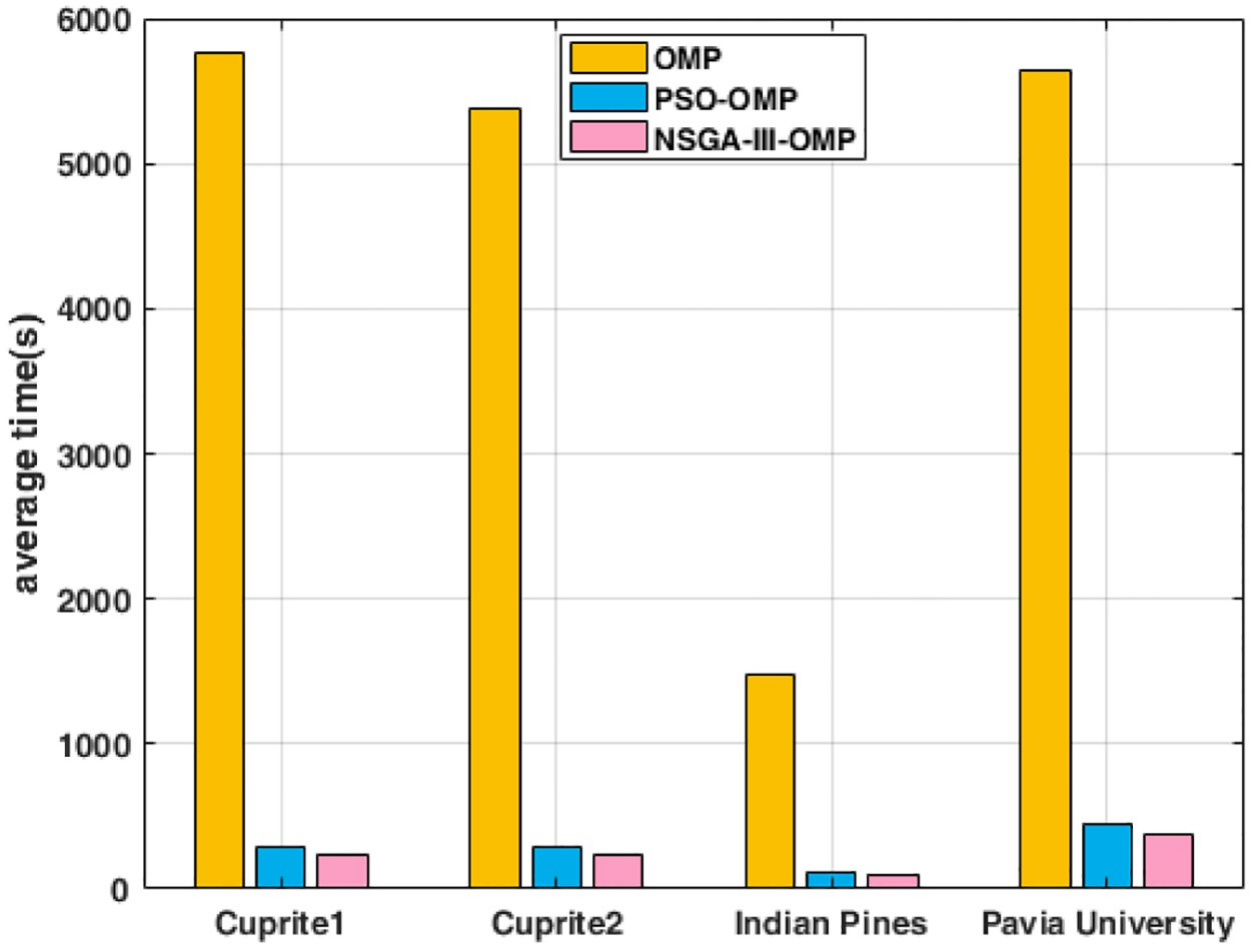
Reconstructed average SAD using OMP, PSO-OMP and NSGA-III-OMP for four datasets.

The runtime of three decomposition algorithms is shown in Fig 10. From the histogram, the evolution algorithms, PSO-OMP and NSGA-III-OMP, can greatly improve the efficiency of optimal atoms matching. In terms of efficiency, NSGA-III-OMP has a slight advantage over PSO-OMP. Compared with OMP, the proposed NSGA-III-OMP can increase the calculation speed by an order of magnitude.

### Reconstruction image comparison

**Fig 11:**
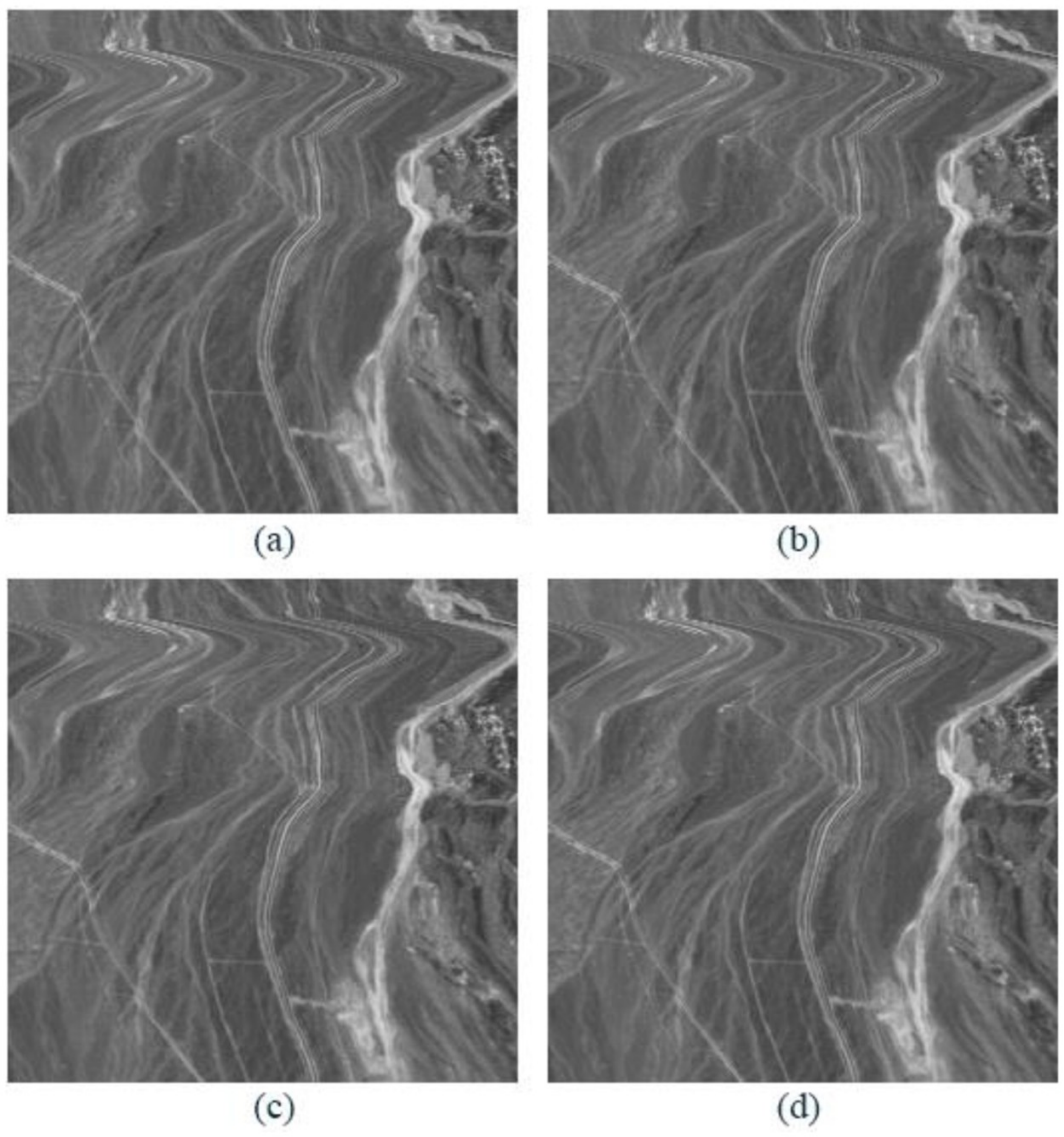
Comparison between original image and reconstructed images of Cuprite1. (a) Original image. (b) Reconstructed image by OMP. (c) Reconstructed image by PSO-OMP. (d) Reconstructed image by NSGA-III-OMP.

As compared to other metrices, a viewer’s preference for pleasant visual quality is more relevant. After the data set Cuprite1 is sparsely decomposed, the comparison between the reconstructed image and the original image is shown in Fig 11. The figure shows the original image and the 40th band of the reconstructed image. The reconstructed PSNR of OMP algorithm, PSO-OMP algorithm and NSGA-III-OMP algorithm can reach 40.11dB, 40.15dB and 42.60dB respectively.

**Fig 12:**
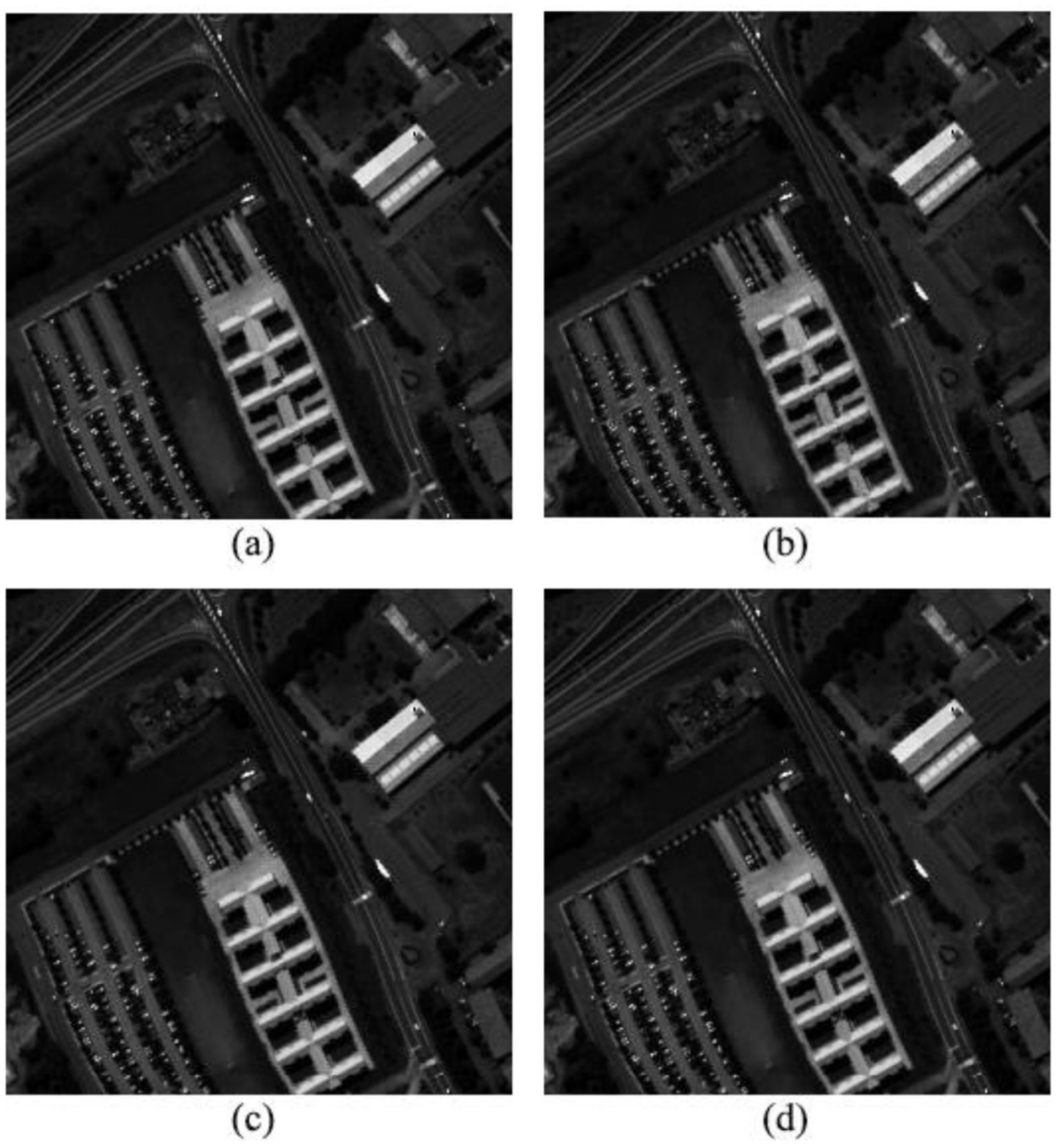
Comparison between original image and reconstructed images of Pavia University. (a) Original image. (b) Reconstructed image by OMP. (c) Reconstructed image by PSO-OMP. (d) Reconstructed image by NSGA-III-OMP.

The comparison between the reconstructed image and the original image of Pavia University is shown in Fig 12. The figure shows the original image and the 40th band of the reconstructed image. The reconstructed PSNR of OMP algorithm, PSO-OMP algorithm and NSGA-III-OMP algorithm can reach 39.66dB, 42.96dB and 46.38dB respectively.

Seen from these reconstruction images, the reconstructed image can well describe the detailed features of the original image, which fully demonstrates that the NSGA-III-OMP algorithm can find the optimal atoms by using the evolution process of the chromosome, complete the high-precision sparse decomposition of the image, and fully demonstrate the effectiveness of the algorithm.

## Conclusions

Analyzing the characteristics of hyperspectral images, a sparse decomposition strategy based on NSGA-III-OMP is proposed. The algorithm utilizes the reference point non-dominated sorting genetic method to solve the constructed multi-objective sparse decomposition optimization model. The algorithm uses the chromosomes of the genetic algorithm to simulate the atom matching process in OMP, and explores the selection, crossover and mutation operators to search the optimal atom. The influence of the population size, the maximum evolutionary generation and the decomposition times on the performance of the algorithm is studied, and reasonable parameters are set in the following experiments. Performance comparisons between proposed NSGA-III-OMP algorithm and other algorithms demonstrate that, our proposed algorithm can effectively improve the reconstruction accuracy and computational efficiency of sparse decomposition.

## Supporting information

S1 Table Basic condition of four hyperspectral datasets used in experiments. (DOCX)

S1 Dataset Band-based PSNR. (XLSX)

S2 Dataset Band-based SSIM. (XLSX)

S3 Dataset Vector-based SNR. (XLSX)

S4 Dataset Vector-based SAD. (XLSX)
