# Supplementary material for: Hyperspectral image compressed processing: Evolutionary multi-objective optimization sparse decomposition
Source: PLoS One. 2022 Apr 29;17(4):e0267754. doi: 10.1371/journal.pone.0267754 (PMC9053777; doi:10.1371/journal.pone.0267754)
Supplement: S1 Table — (DOCX) [file pone.0267754.s001.docx]

**S1 Table. Basic condition of four hyperspectral datasets used in experiments.**

| **Dataset** | **Original bands** | **Original image size** | **Available bands ( removing the water absorption and noisy bands )** | **Cropped image size (spatially cropping for computation convenience)** |
| --- | --- | --- | --- | --- |
| **Cuprite1** | 224 | 614×512 | 188 | 256×256 |
| **Cuprite2** | 224 | 614×512 | 188 | 256×256 |
| **Indian Pines** | 220 | 145×145 | 200 | 128×128 |
| **Pavia University** | 115 | 610×340 | 103 | 256×256 |

The Airborne Visible Infrared Imaging Spectrometer (AVIRIS) collected the Cuprite1 and Cuprite2 images. The sensor collects 224 spectral bands between 0.4 and 2.5m, with a half-maximum of 10 nm and spatial resolution of 20m per pixel. Due to water absorption or noisy, several bands were removed from the study, leaving a total of 188 bands for the experiments.

AVIRIS in northwest Indiana acquired the Indian Pines photos, which have a spatial resolution of 145 by 145 pixels and 220 bands with a wavelength range of 0.4 to 2.5m. Similarly, the bands including the water absorption area have been removed, leaving 200 bands open.

The images obtained by the Reflective Optics System Imaging Spectrometer (ROSIS) at Pavia University comprise 115 bands and a spatial resolution of 1.3 metre per pixel. The 12 noisiest bands in this dataset should be eliminated before analysis, leaving 103 bands in the trials.

The Pines photos are divided into 128 by 128 pixels to avoid impacting the reconstruction algorithm's performance, whereas the other datasets are represented in 256 by 256 pixels.
